# Hypereosinophilic syndrome masquerading as a myocardial infarction causing decompensated heart failure

**DOI:** 10.1186/1471-2261-13-75

**Published:** 2013-09-21

**Authors:** Joanna Lim, Alexander Sternberg, Nathan Manghat, Steve Ramcharitar

**Affiliations:** 1 Wiltshire Cardiac Centre, Great Western Hospitals NHS Foundation Trust, Marlborough Road, Swindon SN3 6BB, UK; 2 Great Western Hospital, Swindon SN3 6BB, UK; 3 Bristol Heart institute, Bristol BS2 8HW, UK

## Abstract

**Background:**

An 81 year old female patient diagnosed with a chronic low grade hypereosinophilic syndrome presented with angina and dyspnoea.

**Case presentation:**

She was managed for a non-ST elevated myocardial infarction since her troponin levels were elevated. On day 5, she suffered an acute clinical deterioration with type I respiratory failure and cardiogenic shock, accompanied by deterioration in left ventricular systolic function demonstrated on echocardiography, and this coincided with a marked rise in eosinophil count. Secondary causes of eosinophilia were excluded permitting a diagnosis of Hypereosinophilic Syndrome (HES) to be made. Coronary angiography revealed unobstructed arteries. Supportive treatment for heart failure included diuretic and inotropes but she dramatically improved both clinically and echocardiographically upon commencement of high dose steroids and hydroxycarbamide. Cardiac magnetic resonance imaging (CMR) demonstrated diffuse, shallow endomyocardial enhancement with late gadolinium, consistent with a diagnosis of eosinophilic myocarditis.

**Conclusion:**

Hypereosinophilic Syndrome can masquerade as a myocardial infarction causing decompensated heart failure. Early recognition and treatment with steroids can improve outcome.

## Background

We present an interesting case of hypereosinophilic syndrome (HES) causing decompensated NYHA class IV heart failure in a patient being managed for a Non-ST elevated myocardial infarct. HES is diagnosed when there is a sustained absolute eosinophil count (AEC) greater than 1.5 × 10^9^ eosinophils per litre for more than 6 months without an identifiable aetiology in patients with signs and symptoms of end organ involvement [1]. The case illustrates the myriad of investigations needed for the diagnosis and the dilemma associated with treatment during the diagnostic work-up as delays can lead to rapid clinical deterioration. These investigations are necessary since secondary causes of eosinophilia can be identified in a proportion of cases that would have otherwise been classified as idiopathic hypereosinophilic syndrome and these may require more targeted therapies. This case also demonstrates the diagnostic potential of cardiac magnetic resonance imaging (CMR) where characteristic myocardial imaging changes can be still apparent months after discharge to confirm the diagnosis and so avoid risky invasive biopsies in the acute setting [2,3].

## Case presentation

An 81 year old South Indian female presented with a two week history of angina, mild dyspnoea, fevers and night sweats. Over the past seven years she had had regular haematology surveillance for a chronic low grade eosinophilia of uncertain aetiology. Other co-morbidities were salbutamol controlled asthma; type 2 diabetes and hypercholesterolaemia managed with metformin 500 mg OD and rosuvastatin 5 mg OD respectively.

At presentation she was afebrile with a heart rate of 84 beats per minute and a blood pressure of 97/60 mmHg. There were no clinical signs of congestive cardiac failure but a grade 2/6 pansystolic murmur was noted at the apex. Respiratory, abdominal and neurological examinations were all normal.

**Figure 1:**
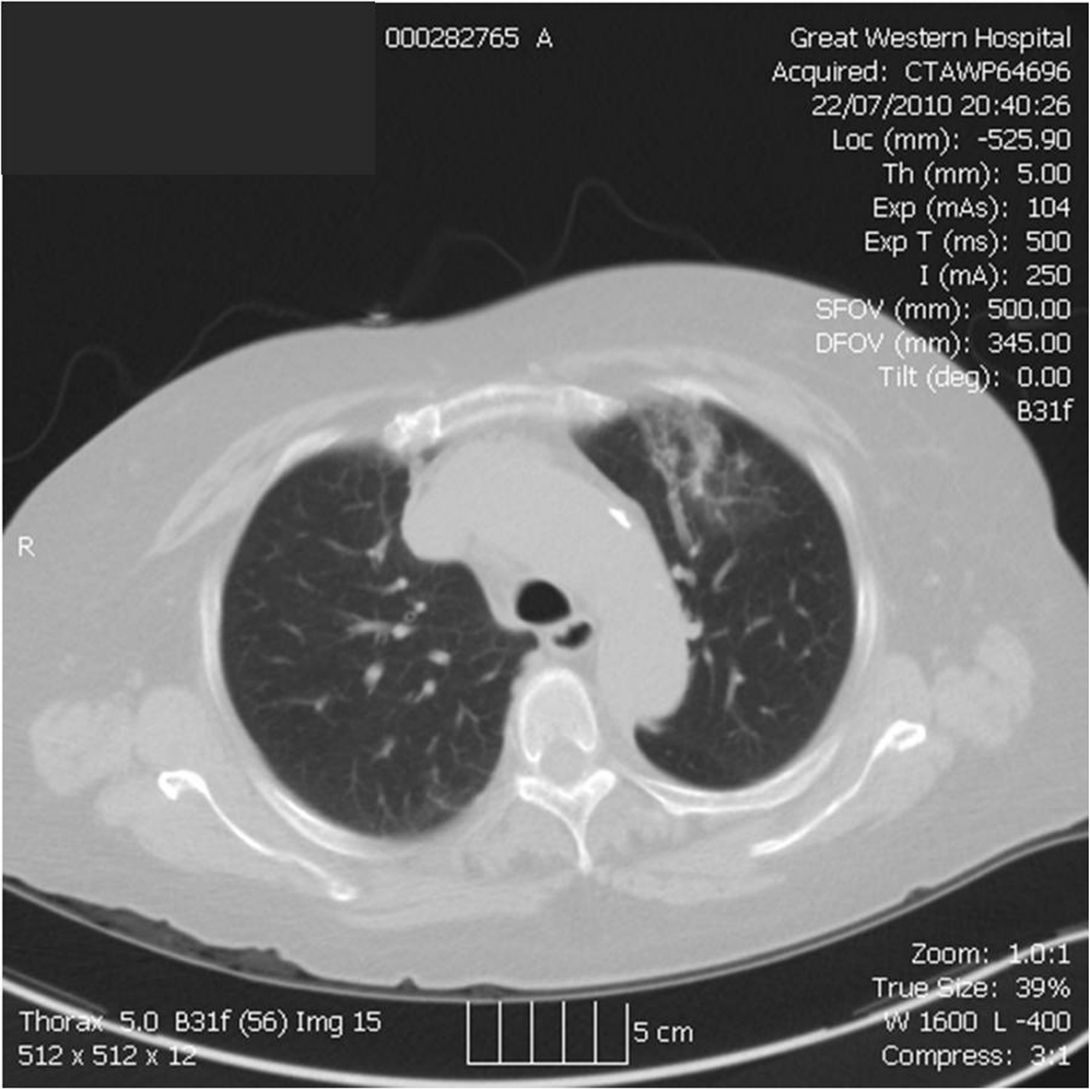
Computed tomography showing non-specific parenchymal distortion in the upper lobe of the left lung.

Her electrocardiogram was normal but her abnormal chest radiograph prompted a thoracic computed tomography which showed non-specific parenchymal distortion in the upper lobe of the left lung (Figure 1); there was no heart failure. Routine biochemistry showed an elevated Troponin I level of 8.5 ng/ml (normal range <0.1 ng/mL). Renal, liver and clotting profiles were all normal. Importantly, she had a significant leucocytosis (total white cells of 27.6 × 10^9^/litre) with a marked eosinophilia (17.39 × 10^9^/litre), well above her chronic low grade eosinophilia (0.45-2.12 × 10^9^/litre). Cellular morphology was normal on blood film.

Cardiac echocardiography demonstrated mild left ventricular hypertrophy and mild left ventricular systolic impairment with akinesia of the basal inferior wall and dyskinesia of the anterior septum. There was moderate tricuspid regurgitation and moderate pulmonary hypertension (pulmonary artery pressure of 53 mmHg. On the basis of her elevated troponin and echocardiographic findings, she was managed for non-ST elevation myocardial infarction (NSTEMI) with aspirin 75 mg OD, clopidogrel 75 mg OD and fondaparinux 2.5 mg OD whilst pending angiography.

**Figure 2:**
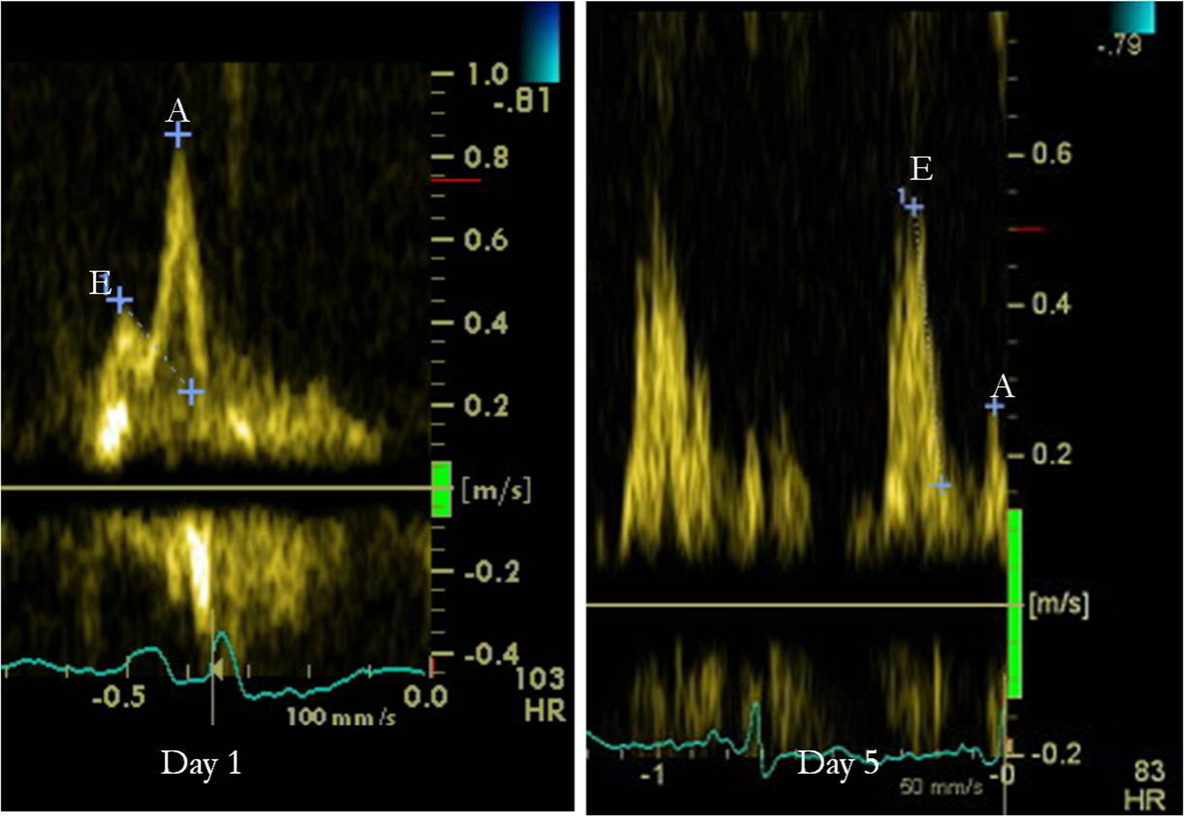
Echocardiogram mitral inflow pulsed wave Doppler showing reversal of E/A ratio indicating a restrictive pattern of filling at day 5.

On day 5 the patient suddenly decompensated with gross heart failure and cardiogenic shock requiring intubation and aggressive intensive care therapy. Her chest radiograph showed marked pulmonary oedema, troponin I remained elevated at 7.8 ng/ml. Arterial blood gas demonstrated a Type 1 respiratory failure with a pH = 7.35, pCO_2_ = 4.1, PO_2_ = 9.5, HCO_3_ = 16.6, BE = -7.7 and Lactate = 9.99. C-Reactive Protein (CRP) was elevated at 50 mg/l. An urgent repeat echocardiogram showed worsened moderate to severe systolic impairment. Interestingly measurement of mitral inflow pulsed wave Doppler showed reversal of E/A ratio which compares the peak velocity of early passive diastolic filling of the ventricle (E wave) to peak velocity of active filling of the ventricle due to atrial contraction (A wave). The normal E/A ratio is 1-2. The patient’s initial E/A ratio at presentation was 0.53 suggestive of mildly abnormal relaxation. At the time of deterioration on day 5, the ratio increased to 2.5 indicating a restrictive pattern of filling (Figure 2).

**Figure 3:**
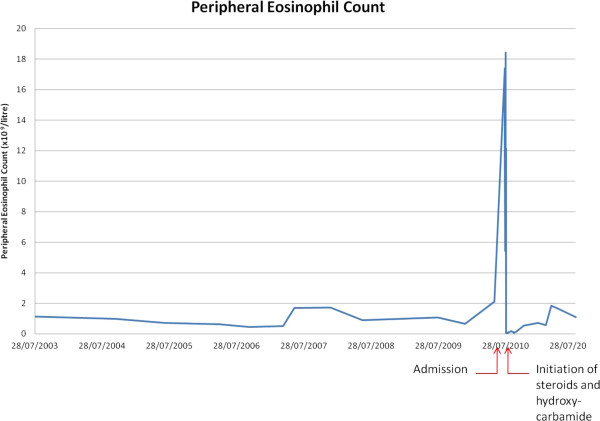
Dramatic reduction in Peripheral Eosinophil Count achieved with steroids and hydroxycarbamide.

Despite cardiac failure therapy comprising intravenous inotropes and diuretics the patient failed to improve clinically. Notably her deterioration seemed to coincide with a peak eosinophilia of 18.44 × 10^9^/litre (Figure 3). Following haematology and microbiology consults oral hydroxycarbamide 1 g OD and high dose intravenous methyprednisolone 500 mg OD were commenced and view of her ethnicity, ivermectin administered to cover potential nematode reactivation.

Concomitantly, secondary causes of eosinophilia were investigated by taking into account atopy related to her history of asthma and her recent medical therapy. Invasive helminth infection (Strongyloides, Toxocara and Filaria) were screened with enzyme linked immunosorbant assays (ELISA). Other causes such as Trichinella were excluded by indirect fluorescence antibody test (IFAT). Serology for Toxoplasma and Human Immunodeficiency Virus were negative. Autoantibody screen, cANCA and pANCA were also negative and her immunoglobulin profiles were normal. Sarcoidosis was excluded with a negative serum ACE. Bone marrow aspirate and flow cytometry showed increased numbers of maturing eosinophils but no clonal expansion. Cytogenetic analysis demonstrated normal karyotype, in particular no FIP1L1-PDGFRA gene fusion [4].

On steroid treatment the eosinophilic count dropped dramatically and her clinical state rapidly improved. A repeat echocardiogram on day 14 demonstrated marked improvement in her left ventricular systolic dysfunction (mild-moderate systolic impairment) and her heart failure was now NYHA class I. Standard oral heart failure therapies (ACE-inhibitor, betablocker and diuretics) were optimised, hydroxycarbamide was stopped on day 24 and steroids weaned over an 8 week period.

Subsequent coronary angiography demonstrated normal, unobstructed coronary arteries and the patient was discharged home in an asymptomatic state at day 50.

**Figure 4:**
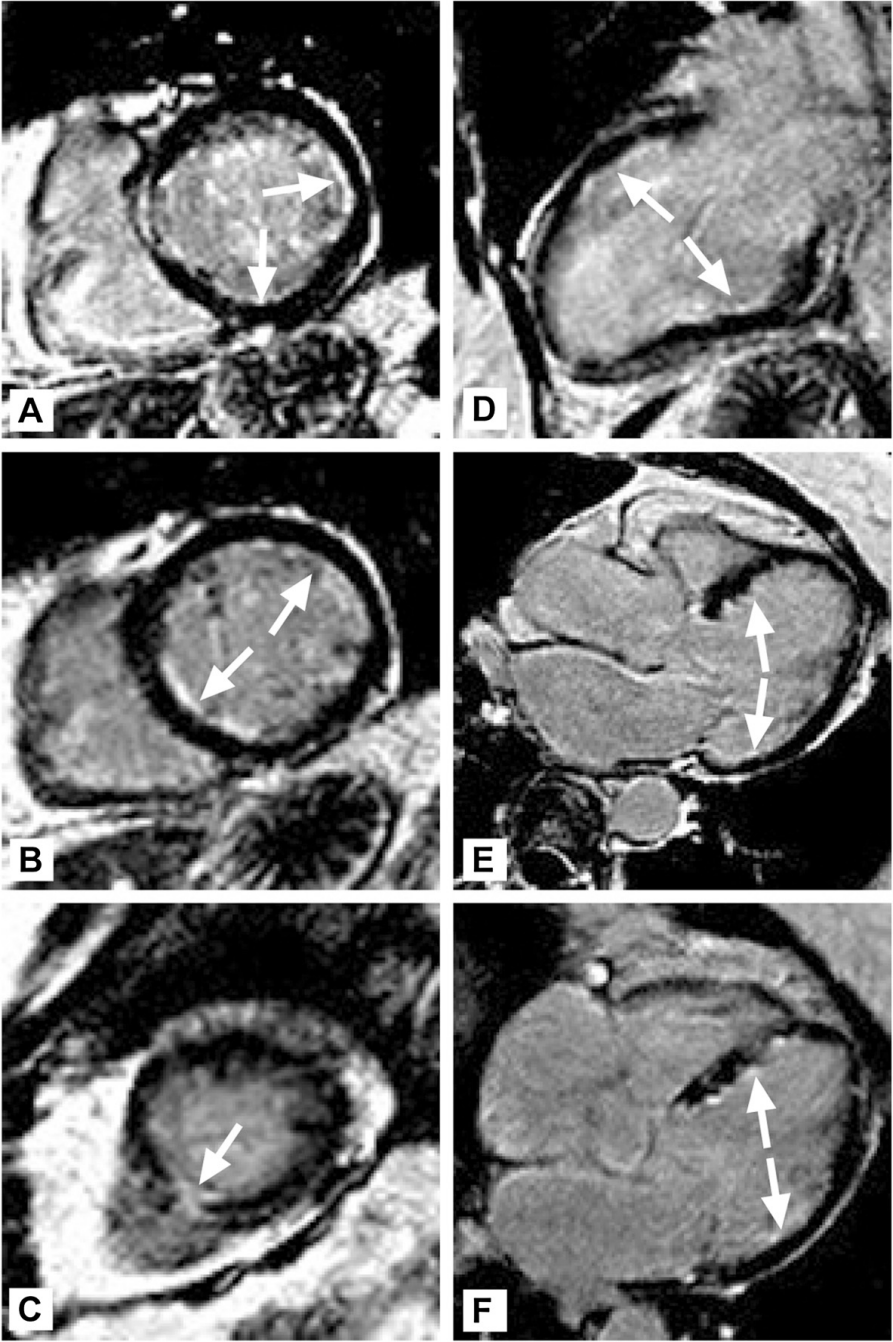
**Panels A-F Cardiac Magnetic Resonance Imaging–Phase sensitive inversion recovery gadolinium delayed enhancement images through the left ventricle are shown in short axis planes (A-C): basal, mid and apical cross-sections, and in long axis planes (D-E): 2-chamber view showing the anterior and inferior walls, 3-chamber showing the antero-septal and infero-lateral walls and 4-chamber showing the infero-septal and antero-lateral walls.** Normal myocardial signal is nulled and therefore appears black. The white arrows point to the multi-territorial regions of shallow subendocardial hyperenhancement; **C**–also demonstrates a focus of more transmural inferoapical involvement. Features are in keeping with hypereosinophilic endomyocarditis.

An outpatient cardiac magnetic resonance imaging (CMR) at 3 months clinic follow-up showed a pattern typical of eosinophilic endomyocarditis with post-inflammatory scarring [5] (Figure 4).

## Discussion

Hyperosinophilic syndrome (HES) is defined as a primary idiopathic eosinophilia with >1.5 × 10^9^ eosinophils per litre of peripheral blood for at least 6 months with evidence of end organ dysfunction. This end organ damage in the myocardium (endocardial mural fibrosis) was first noted by Loeffler as ‘endocarditis parietalis fibroelastica’ [6]. This should not however be confused with ‘Loeffler’s syndrome’ which comprises hypereosinophilia with pulmonary infiltrates and sparing of the myocardium [7]. The cardiotoxic effects of the eosinophils secreting eosinophil granulated proteins (EGP) results in a spectrum of clinical manifestations which includes acute pericarditis, myocarditis or endocarditis (‘necrotic’ phase, mean duration 5.5 weeks); formation of thrombi adjacent to the injured endocardium (‘thrombotic’ phase, mean duration 10 months); and localised or generalised replacement fibrosis (‘fibrotic’ phase, mean duration 24.5 months) leading to restrictive heart failure [8].

Any delay in the treatment can result in significant co-morbidity from restrictive heart failure. Thus solely treating the heart failure without simultaneously addressing the eosinophilia can have significant clinical consequences [9]. The dilemma facing many clinicians in such a scenario is the temptation to with hold steroids whilst awaiting the results from the numerous investigations needed to exclude secondary causes of the eosinophilia. Also there is a risk that steroids can worsen cardiac failure. At present there are no randomized controlled trials to support the use of steroids only anecdotal reports [10]. Other therapies that include hydroxycarbamide, interferon alpha, cytotoxic chemotherapy and more recently, targeted molecular therapy with imatinib mesylate, a tyrosine kinase inhibitor have all been used with some success [11]. But again there is often a delay in implementing the more complex treatment (imatinib requires karyotyping for FIPIL1-PDGFRA mutation) and moreover some therapies can further deteriorate left ventricular systolic function as the eosinophils lyse and degranulate [11].

In the acutely ill patient the ‘gold standard’ endomyocardial biopsy (EMB) can have significant access complications (bleeding, pneumothorax, infection). Moreover biopsies can cause ventricular perforation, arrhythmia and conduction abnormality, and the significant sampling error related to patchy or focally distributed myocarditis together with inter-interpreter variability can also limit its effectiveness [3]. Consequently, there is now an emerging role for the use of CMR to guide the EMB or to make the diagnosis [5]. A bright, diffuse subendocardial enhancement is typically seen on late gadolinium enhancement, along with low signal structures representing intra-cavitary thrombi. CMR can also delineate the precise extent of the disease, facilitate longer term follow-up and assess the response to treatment [12]. In our case it proved to be a crucial tool in confirming the diagnosis as the morphological changes were still apparent 3 months following discharge even though the patient was asymptomatic on medical treatment [13].

## Conclusion

Hypereosinophilic Syndrome can masquerade as a myocardial infarction causing decompensated heart failure. Early recognition and treatment with steroids can improve outcome and should be started as soon as possible when secondary causes of eosinophilia are excluded. CMR can be used to delineate the precise extent of the disease, facilitate longer term follow-up and to assess the response to treatment.

## Consent

Written informed consent was obtained from the patient for publication of this case report and any accompanying images. A copy of the written consent is available for review by the Editor-in-Chief of this journal.

## Abbreviations

HES: Hypereosinophilic syndrome; AEC: Absolute eosinophil count; NYHA: New York Heart Association; CMR: Magnetic resonance imaging; OD: Once a day; ANCA: Anti-neutrophil cytoplasmic antibodies; ACE: Angiotensin-converting enzyme; EMB: Endomyocardial biopsy.

## Competing interests

All authors declare that they have no competing interests.

## Authors’ contributions

JL was involved in the conception, design and writing. AS wrote part of the manuscript and commented on the haematological investigations. NM drafted the manuscript and revised it critically for important intellectual content. SR conceived, designed, wrote and supervised the construction of the publication and was responsible for the patient’s clinical care. All authors (JL, AS, NM and SR) have given final approval of the version to be published.

## Pre-publication history

The pre-publication history for this paper can be accessed here:

http://www.biomedcentral.com/1471-2261/13/75/prepub
